# Genome sequence and description of *Nesterenkonia massiliensis* sp. nov. strain NP1^T^

**DOI:** 10.4056/sigs.5631022

**Published:** 2014-04-01

**Authors:** Sophie Edouard, Senthil Sankar, Nicole Prisca Makaya Dangui, Jean-Christophe Lagier, Caroline Michelle, Didier Raoult, Pierre-Edouard Fournier

**Affiliations:** 1 Aix-Marseille Université, Faculté de médecine, Marseille, France.; 2 Special Infectious Agents Unit, King Fahd Medical Research Center, King Abdulaziz University, Jeddah, Saudi Arabia

**Keywords:** *Nesterenkonia massiliensis*, genome, culturomics, taxono-genomics

## Abstract

*Nesterenkonia massiliensis* sp. nov., strain NP1^T^, is the type strain of *Nesterenkonia massiliensis* sp. nov., a new species within the genus *Nesterenkonia*. This strain, whose genome is described here, was isolated from the feces of a 32-year-old French woman suffering from AIDS and living in Marseille. *Nesterenkonia massiliensis* is a Gram-positive aerobic coccus. Here, we describe the features of this bacterium, together with the complete genome sequencing and annotation. The 2,726,371 bp long genome (one chromosome but no plasmid) contains 2,663 protein-coding and 51 RNA genes, including 1 rRNA operon.

## Introduction

*Nesterenkonia massiliensis* strain NP1**^T^** (= CSUR P244 = DSM 26221) is the type strain of *N. massiliensis* sp. nov. This bacterium is a Gram-positive, non-spore-forming, aerobic and motile coccus that was isolated from the fecal flora of AIDS-infected French female living in Marseille, France, as part of a “culturomics” study aiming at cultivating as many of the bacterial species in human feces as possible [1,2].

Taking advantage of the availability of more than 12,000 bacterial genome sequences [3], we recently proposed to use genomic properties in combination with phenotypic characteristics for the taxonomic classification of *Bacteria* [4-34].

Herein, we present a summary classification and a set features for *Nesterenkonia massiliensis* sp. nov., strain NP1**^T^** (CSUR= P244 = DSM 26221), including the description of its complete genome and annotation. These characteristics support the circumscription of the species *Nesterenkonia massiliensis*.

The genus *Nesterenkonia* was first described by Stackebrandt *et al*. in 1995 [35]. This genus belongs to the family *Micrococcaceae* within the phylum *Actinobacteria*, and is most closely related to the genera *Micrococcus, Arthrobacter and Kocuria* [35]. The *Nesterenkonia* genus includes Gram-positive, non spore-forming, aerobic, mesophilic bacteria that may be halotolerant or halophilic. Currently, the genus *Nesterenkonia* includes 12 species with validly published names [36]. Members of the genus *Nesterenkonia* are ubiquitous bacteria, which have been isolated from various environments including hypersaline soil and lakes, soda lakes, sea food, and paper and cotton pulp mills [36-40]. However, prior to our study, *Nesterenkonia* species had not been reported in humans, with the exception of DNA sequences from *Nesterenkonia* sp. detected in the gut microbiota of patients with chronic kidney diseases [41].

## Classification and features

**Figure 1:**
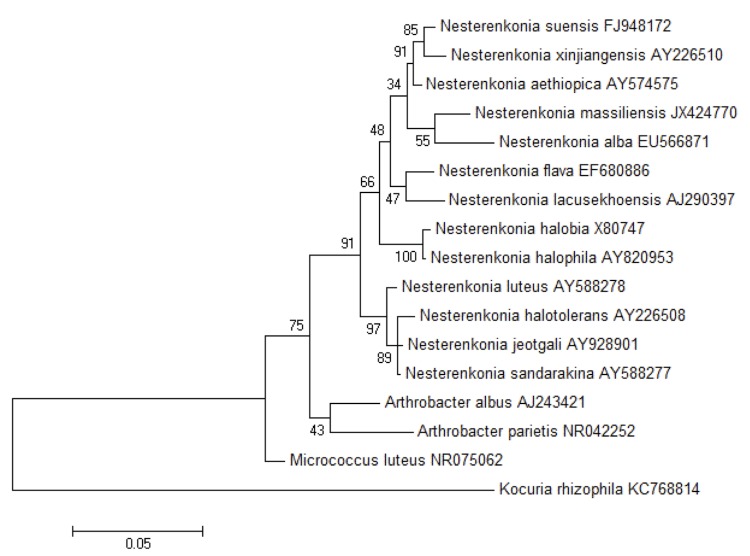
Consensus phylogenetic tree highlighting the position of *Nesterenkonia massiliensis* strain NP1**^T^** relative to other type strains within the *Nesterenkonia* genus. Genbank accession numbers are indicated for each species. Sequences were aligned using CLUSTALW and phylogenetic inferences obtained using the maximum-likelihood method in the MEGA software package. Numbers at the nodes are percentages of bootstrap values from 500 replicates that support the node. *Micrococcus luteus* was used as the outgroup. The scale bar represents a 5% nucleotide sequence divergence.

A stool sample was collected from a Caucasian, AIDS-infected, 32-year-old French woman living in Marseille, France. The patient gave an informed and signed consent. This study and the assent procedure were approved by the Ethics Committee of the Institut Fédératif de Recherche IFR48, Faculty of Medicine, Marseille, France under agreement number 09-022. The fecal sample was preserved at -80°C after collection. Strain NP1^T^ (Table 1) was first isolated in March 2012 by cultivation on Columbia agar (BioMerieux, Marcy l’Etoile, France) under aerobic conditions after 14 days of preincubation of the stool sample with addition of 5ml of sheep rumen in blood bottle culture. The strain exhibited a 96.7% 16S rRNA nucleotide sequence identity with *N. alba* [51], the phylogenetically most closely related *Nesterenkonia* species with standing in nomenclature (Figure 1). This value was lower than the 98.7% 16S rRNA gene sequence threshold recommended by Stackebrandt and Ebers to delineate a new species without carrying out DNA-DNA hybridization [52].

**Table 1:** Classification and general features of *Nesterenkonia massiliensis* strain NP1 **^T^** according to the MIGS recommendations [42].

**MIGS ID**	**Property**	**Term**	**Evidence code^a^**
	Current classification	Domain *Bacteria*	TAS [43]
		Phylum *Actinobacteria*	TAS [44]
		Class *Actinobacteria*	TAS [45]
		Order *Actinomycetales*	TAS [45-48]
		Family *Micrococcaceae*	TAS [45-47,49]
		Genus *Nesterenkonia*	TAS [35,39,45]
		Species *Nesterenkonia massiliensis*	IDA
		Type strain: NP1	IDA
	Gram stain	Positive	IDA
	Cell shape	Cocci	IDA
	Motility	Motile	IDA
	Sporulation	Nonsporulating	IDA
	Temperature range	Mesophile	IDA
	Optimum temperature	Unknown	IDA
MIGS-6.3	Salinity	Halophilic	IDA
MIGS-22	Oxygen requirement	Aerobic	IDA
	Carbon source	Unknown	NAS
	Energy source	Unknown	NAS
MIGS-6	Habitat	Human gut	IDA
MIGS-15	Biotic relationship	Free living	IDA
MIGS-14	Pathogenicity	Unknown	
	Biosafety level	2	
	Isolation	Human feces	
MIGS-4	Geographic location	Marseille, France	IDA
MIGS-5	Sample collection time	March 2012	IDA
MIGS-4.1	Latitude Longitude	43.296482 5.36978	IDA
MIGS-4.3	Depth	Surface	IDA
MIGS-4.4	Altitude	0 m above sea level	IDA

^a^Evidence codes - IDA: Inferred from Direct Assay; TAS: Traceable Author Statement (i.e., a direct report exists in the literature); NAS: Non-traceable Author Statement (i.e., not directly observed for the living, isolated sample, but based on a generally accepted property for the species, or anecdotal evidence). These evidence codes are from the Gene Ontology project [50]. If the evidence is IDA, then the property was directly observed for a live isolate by one of the authors or an expert mentioned in the acknowledgements.

**Figure 2:**
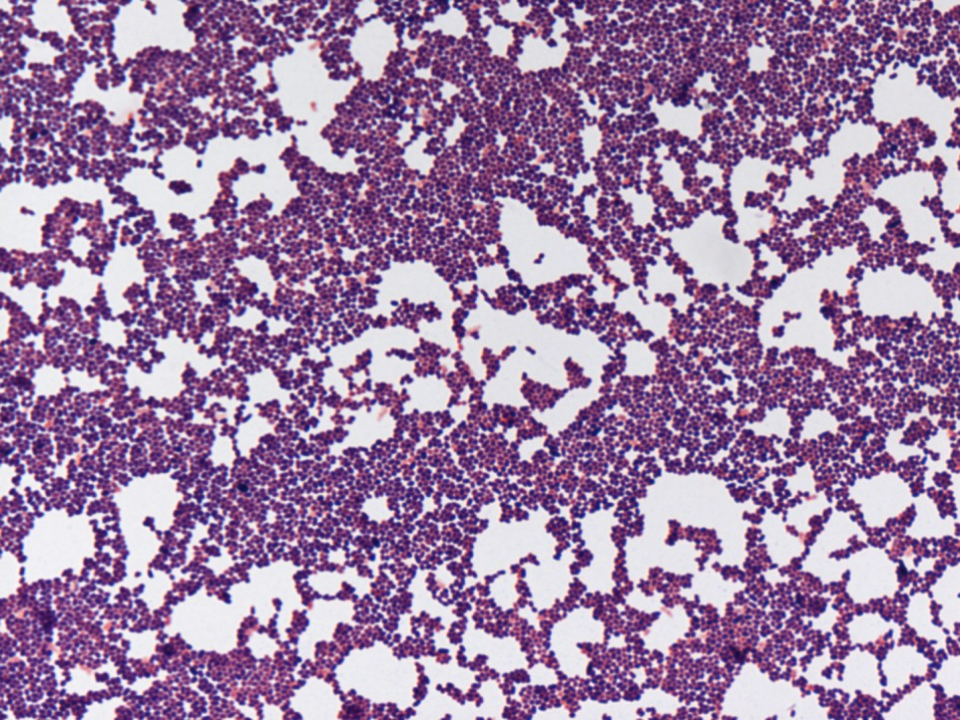
Gram-stain of *Nesterenkonia massiliensis* strain NP1**^T^**

**Figure 3:**
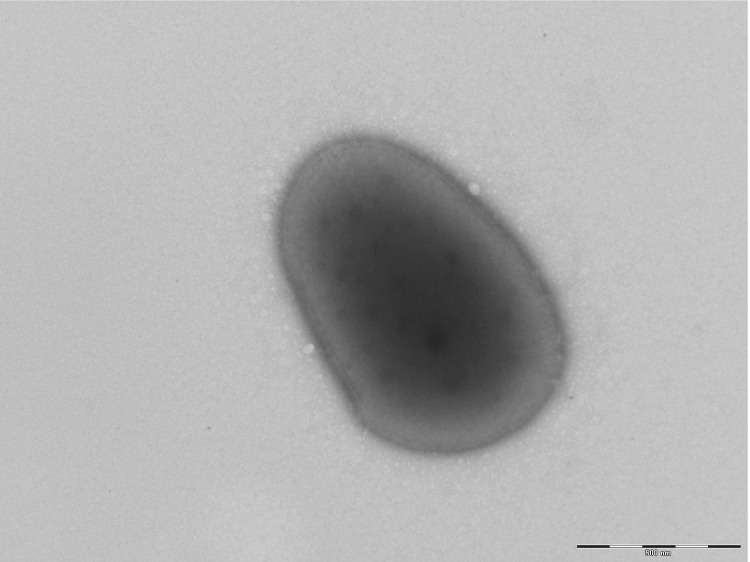
Transmission electron micrograph of *N. massiliensis* strain NP1**^T^**, made using a Morgani 268D (Philips) at an operating voltage of 60kV. The scale bar represents 500 nm.

Four different growth temperatures (25, 30, 37, 45°C) were tested. Growth was observed between 25 and 45°C on blood-enriched Columbia agar (BioMerieux), with optimal growth occurring at 37°C after 24 hours of incubation. Colonies were dark yellow and 1 mm in diameter. Growth of the strain was tested under anaerobic and microaerophilic conditions using GENbag anaer and GENbag microaer systems, respectively (BioMerieux), and under aerobic conditions, with or without 5% CO_2_. Optimal growth was obtained under aerobic conditions, but weak growth occurred in a microaerophilic atmosphere. No growth was observed under anaerobic conditions. Bacterial cells were Gram-positive (Figure 2), non-endospore-forming, and motile cocci. Cells grown on agar had a mean diameter and length of 0.67 μm and 1.4 μm, respectively (Figure 3).

Strain NP1**^T^** exhibited catalase but no oxidase activity. Using an API 20NE strip (BioMerieux), negative reactions were obtained for nitrate reduction, urease, indole production, glucose fermentation, arginine dihydrolase, β-galactosidase, glucose, arabinose, mannose, mannitol, N-acetyl-glucosamine, maltose, gluconate, caprate, adipate, malate, citrate, phenyl-acetate assimilation and cytochrome oxidase. Substrate oxidation and assimilation were examined with an API 50CH carbohydrate fermentation strip (BioMerieux) at 37°C. Positive reactions were observed for D-glucose, D-fructose, D-saccharose, ribose, mannose, mannitol, D-trehalose and L-rhamnose. No reaction was observed for esculin, salicin, D-cellobiose and gentiobiose. *N. massiliensis* is susceptible to amoxicillin, imipenem, rifampin, ciprofloxacin, gentamicin, doxycycline and vancomycin but resistant to trimethoprim/sulfamethoxazole and metronidazole. When compared with representative species from the genus *Nesterenkonia*, *N. massiliensis* strain NP1**^T^** differed in cell shape, colony color, motility, optimal growth temperature, and mannitol fermentation (Table 2).

**Table 2:** Differential characteristics of *Nesterenkonia* species*.

	***N. massiliensis***	***N. alba***	***N. flava***	***N. lacusekhoensis***
Cell diameter (µm)	0.7x1.4	0.4-0.6 x 0.8-1.2	0.6-0.8 x1.2- 1.4	0.8-0.9 x 1-1.3
Oxygen requirement	Aerobic	Aerobic	Aerobic	Aerobic
Pigment production	Dark Yellow	White	Yellow	Bright yellow
Gram stain	cocci	Short rods	Short rods	Short rods
Optimal temperature (^o^C)	37°C	42	40-42	27-33.5
Salt requirement NaCl tolerance (%)	0-3	0-6	0-10	0-15
Motility	+	-	-	-
Endospore formation	-	-	-	-
H2S production	-	-	-	W
Indole production	-	-	-	-
ONPG test*	NA	+	-	NA
Citrate test	NA	-	-	W
Voges–Proskauer reaction	NA	-	-	-
Production of				
Catalase	+	+	+	+
Oxidase	-	-	-	-
Nitrate reductase	-	-	-	-
Urease	-	-	-	NA
Β-galactosidase	-	NA	-	NA
Acid from				
L-arabinose	-	+	+	NA
Mannose	-	-	+	+
Mannitol	+	NA	-	-
Sucrose		+	+	+
D-glucose	+	+	+	+
D-fructose	+	-	+	+
D-maltose	+	+	+	+
D-lactose	-	-	-	-
D-Galactose	-	-	-	-
Trehalose	-	W	-	+
D-Xylose	-	-	-	-
Hydrolysis of:				
Starch	-	-	+	-
Gelatin	-	+	+	-
G+C content (mol%)	62.47	60.2	65.5	66.1
Habitat	Human gut, France	Black liquor treatment system of a cotton pulp mill, China	Paper mill effluent, China	Hypersaline lake, East Antarctica

+, Positive; –, negative; W, weak reaction; NA, not available.

* *N. massiliensis* strain NP^T^, *N. alba* strain DSM 19423, *N. flava* strain JCM 14814^T^, *N. lacusekhoensis* strain DSM 12544

**Figure 4:**
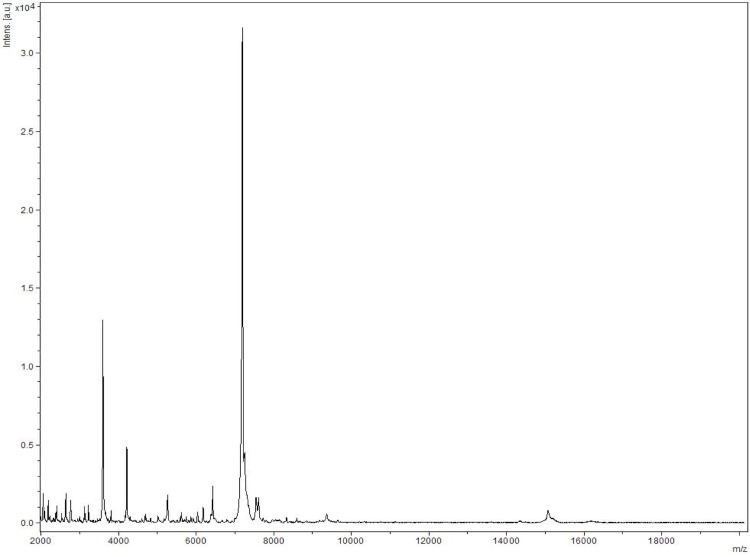
Reference mass spectrum from *Nesterenkonia massiliensis* strain NP1^T^. Spectra from 12 individual colonies were compared and a reference spectrum was generated.

**Figure 5:**
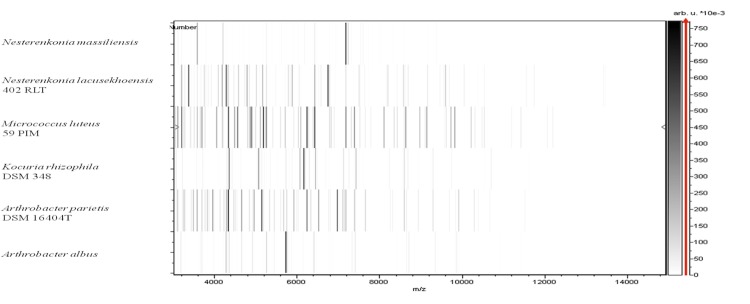
Gel view comparing *N. massiliensis* strain NP1**^T^**, *N. lacusekhoensis* and other species belonging to the family *Micrococcaceae*. The gel view displays the raw spectra of loaded spectrum files arranged as a pseudo-electrophoretic gel. The x-axis records the m/z value. The left y-axis displays the running spectrum number originating from subsequent spectra loading. The peak intensity is expressed by a grey scale scheme. The grey scale on the right y-axis indicates the relative peak intensity in arbitrary units. Species names are on the left.

Matrix-assisted laser-desorption/ionization time-of-flight (MALDI-TOF) MS protein analysis was carried out as previously described [53] using a Microflex spectrometer (Brüker Daltonics, Leipzig, Germany). Twelve individual NP1**^T^** colonies were deposited on a MTP 384 MALDI-TOF target plate (Brüker). The twelve spectra were imported into the MALDI BioTyper software (version 2.0, Brüker) and analyzed by standard pattern matching (with default parameter settings) against the main spectra of 4,706 bacteria, including 1 spectrum from *N. lacusekhoensis*, the only validly named *Nesterenkonia* species for which a spectrum was available in the BioTyper database. A score enabled the presumptive identification and discrimination of the tested species from those in a database: a score > 2 with a validated species enabled the identification at the species level; and a score < 1.7 did not enable any identification. For strain NP1**^T^**, no significant score was obtained, suggesting that our isolate was not a member of any known species (Figures 4 and 5).

## Genome sequencing information

### Genome project history

The organism was selected for sequencing on the basis of its phylogenetic position, 16S rDNA similarity and phenotypic differences with other members of the genus *Nesterenkonia*, and is part of a “culturomics” study aiming at isolating individually all bacterial species within the human gut flora [1]. It was the third genome of a *Nesterenkonia* species and the first genome of *N. massiliensis* sp. nov. A summary of the project information is shown in Table 3. The Genbank accession number is CBLL000000000 and consists of 141 contigs. Table 3 shows the project information and its association with MIGS version 2.0 compliance [42].

**Table 3:** Project information

**MIGS ID**	**Property**	**Term**
MIGS-31	Finishing quality	High-quality draft
MIGS-28	Libraries used	One 454 paired-end 3-kb library
MIGS-29	Sequencing platforms	454 GS FLX Titanium
MIGS-31.2	Fold coverage	22.63×
MIGS-30	Assemblers	Newbler version 2.5.3
MIGS-32	Gene calling method	Prodigal
	INSDC ID	PRJEB668
	Genbank ID	CBLL000000000
	Genebank Date of release	August 20, 2013
	Gold ID	Gi39432
MIGS-13	Project relevance	Study of human gut microbiome

### Growth conditions and DNA isolation

*N. massiliensis* sp. nov strain NP1**^T^**, (= CSUR P244 = DSM 26221) was grown aerobically on sheep blood-enriched Columbia agar at 37°C. Two petri dishes were spread and the colonies resuspended in 6x100 µl of G2 buffer (EZI DNA Tissue Kit, Qiagen). A first mechanical lysis was performed with glass powder on the Fastprep-24 device (Sample Preparation system, MP Biomedicals, USA) using 2x20 second bursts. DNA was incubated with 2.5 µg/µL of lysozyme for 30 minutes at 37°C and extracted using the BioRobot EZ 1 Advanced XL (Qiagen).The DNA was then concentrated and purified on a Qiamp kit (Qiagen). The DNA concentration measured by the Quant-it Picogreen kit (Invitrogen) on the Genios Tecan fluorometer was 94 ng/µl.

### Genome sequencing and assembly

DNA (5 µg) was mechanically fragmented with a Hydroshear device (Digilab, Holliston, MA, USA) with an enrichment size at 3-4 kb. The DNA fragmentation was visualized through the Agilent 2100 BioAnalyzer on a DNA labchip 7500 with an average size of 3.5 kb. The library was constructed using a 454 GS-FLX Titanium paired-end rapid library protocol. Circularization and nebulization generated a pattern with an average size of 390 bp. After 20 cycles of PCR amplification, the double stranded paired-end library was quantified using the Quant-it Ribogreen kit (Invitrogen) using a Genios Tecan fluorometer at 820 pg/µL. The library concentration equivalence was calculated as 3.86 x 10^9^ molecules/µL. The library was stored at -20°C until further use.

The library was clonally amplified with 1 cpb in two emPCR reactions with the GS Titanium SV emPCR Kit (Lib-L) v2. The yield of the emPCR was 13.67%, within the range of 5 to 20% recommended for the Roche procedure. Approximately 656,601 beads were loaded for a ¼ region on the GS Titanium PicoTiterPlate (PTP Kit 70x75, Roche) and sequenced with the GS Titanium Sequencing Kit XLR70. The run was performed overnight and analyzed on the cluster through the gsRunBrowser and Newbler assemblers (Roche). A total of 176,833 passed filter wells were obtained and generated 60.9 Mb with an average of length of 345 bp. The passed filter sequences were assembled on Newbler with 90% identity and 40 bp as overlap. The final assembly identified 18 scaffolds and 141 large contigs (>1,500 bp), and generated a genome size of 2.69Mb which corresponds to a coverage of 22.63 genome equivalents.

### Genome annotation

Open Reading Frames (ORFs) were predicted using Prodigal [54] with default parameters but the predicted ORFs were excluded if they spanned a sequencing gap region. The predicted bacterial protein sequences were searched against the GenBank database [55] and the Clusters of Orthologous Groups (COG) databases using BLASTP. The tRNAScanSE tool [56] was used to find tRNA genes, whereas ribosomal RNAs were found by using RNAmmer [57] and BLASTn against the GenBank database. Lipoprotein signal peptides and the number of transmembrane helices were predicted using SignalP [58] and TMHMM [59] respectively. ORFans were identified if their BLASTP *E*-value was lower than 1e^-3^ for alignment length greater than 80 amino acids. If alignment lengths were smaller than 80 amino acids, we used an *E*-value of 1e^-5^. Such parameter thresholds have already been used in previous works to define ORFans. Artemis [60] and DNA Plotter [61] were used for data management and visualization of genomic features, respectively. The Mauve alignment tool (version 2.3.1) was used for multiple genomic sequence alignment [62]. To estimate the mean level of nucleotide sequence similarity at the genome level, we used the Average Genomic Identity Of gene Sequences (AGIOS) home-made software [34]. Briefly, this software combines the Proteinortho software [63] for detecting orthologous proteins in pairwise comparisons of genomes, then retrieves the corresponding genes and determines the mean percentage of nucleotide sequence identity among orthologous ORFs using the Needleman-Wunsch global alignment algorithm. As only one genome was available for the genus *Nesterenkonia*, we used genomes from closely related genera for the calculation of AGIOS values. *N. massiliensis* strain NT1^T^ was compared to *Nesterenkonia alba* strain DSM 19423 (GenBank accession number ATXP00000000), *Micrococcus luteus* strain NCTC2665 (CP001628), *Kocuria rhizophila* strain DSM 2048 (AP009152) and *Arthrobacter arilaitensis* strain RE117 (FQ311875).

## Genome properties

**Figure 6:**
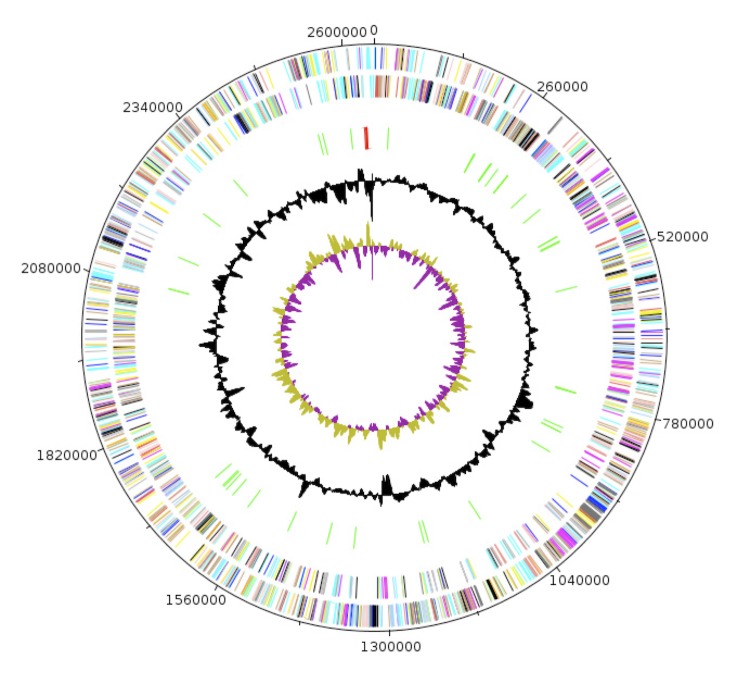
Graphical circular map of the chromosome. From the outside in, the outer two circles show open reading frames oriented in the forward (colored by COG categories) and reverse (colored by COG categories) directions, respectively. The third circle marks the rRNA gene operon (red) and tRNA genes (green). The fourth circle shows the G+C% content plot. The inner-most circle shows GC skew, purple indicating negative values and olive, positive values.

**Figure 7:**
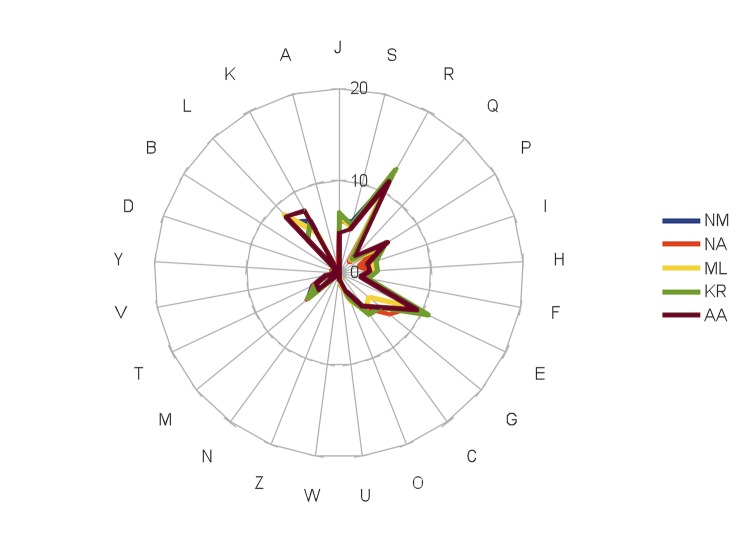
Distribution of functional classes of predicted genes on the chromosomes of *Nesterenkonia massiliensis* (NM), *Nesterenkonia alba* (NA), *Micrococcus luteus* (ML), *Kocuria rhizophila* (KR) and *Arthrobacter arilaitensis* (AA) according to the COG category. For each genome, we indicated the percentage of each gene category.

The genome of *N. massiliensis* strain NP1^T^ is 2,726,371bp long (1 chromosome, but no plasmid) with a 62.47% G+C content (Table 4, Figure 6, Figure 7). Of the 2,714 predicted genes, 2,663 were protein- coding genes, and 51 were RNAs. Three rRNA genes (one 16S rRNA, one 23S rRNA and one 5S rRNA) and 48 predicted tRNA genes were identified in the genome. A total of 1,962 genes (73.68%) were assigned a putative function. One hundred and ninety-nine genes were identified as ORFans (7.47%). The remaining genes were annotated as hypothetical proteins. The properties and the statistics of the genome are summarized in Table 4. The distribution of genes into COGs functional categories is presented in Table 5 and a comparison is presented in Table 6.

**Table 4:** Nucleotide content and gene count levels of the genome

**Attribute**	Value	% of total^a^
Genome size (bp)	2,726,371	100
DNA coding region (bp)	2,473,018	90.40
DNA G+C content (bp)	1,703,271	62.47
Total genes	2714	100
RNA genes	51	1.88
Protein-coding genes	2663	98.12
Genes with function prediction (Cogs + NR)	1962	73.68
Genes assigned to COGs	1950	73.23
Genes with peptide signals	241	9.05
Genes with transmembrane helices	599	22.49

^a^ The total is based on either the size of the genome in base pairs or the total number of protein coding genes in the annotated genome.

**Table 5:** Number of genes associated with the 25 general COG functional categories

**Code**	**Value**	**%age^a^**	**Description**
J	149	5,60	Translation
A	1	0,04	RNA processing and modification
K	162	6,08	Transcription
L	188	7,06	Replication, recombination and repair
B	1	0,04	Chromatin structure and dynamics
D	20	0,75	Cell cycle control, mitosis and meiosis
Y	0	0	Nuclear structure
V	36	1,35	Defense mechanisms
T	78	2,93	Signal transduction mechanisms
M	112	4,21	Cell wall/membrane biogenesis
N	3	0,11	Cell motility
Z	0	0	Cytoskeleton
W	0	0	Extracellular structures
U	29	1,09	Intracellular trafficking and secretion
O	71	2,67	Posttranslational modification, protein turnover, chaperones
C	129	4,84	Energy production and conversion
G	148	5,56	Carbohydrate transport and metabolism
E	239	8,97	Amino acid transport and metabolism
F	67	2,52	Nucleotide transport and metabolism
H	88	3,30	Coenzyme transport and metabolism
I	73	2,74	Lipid transport and metabolism
P	160	6,01	Inorganic ion transport and metabolism
Q	48	1,80	Secondary metabolites biosynthesis, transport and catabolism
R	293	11,00	General function prediction only
S	142	5,33	Function unknown
_	713	26,77	Not in COGs

^a^The total is based on the total number of protein coding genes in the annotated genome

**Table 6:** Genomic comparison of *N. massiliensis* and 4 other members of the family *Micrococcaceae*^†^.

	*Nesterenkonia massilensis*	*Nesterenkonia alba*	*Micrococcus luteus*	*Kocuria rhizophila*	*Arthrobacter arilaitensis*
*Nesterenkonia massilensis*	**2,663**	1,142	1,132	1,087	1,208
*Nesterenkonia alba*	75.61	**2,351**	1,046	1,033	1,116
*Micrococcus luteus*	70.5	1,046	**2,236**	1,152	1,241
*Kocuria rhizophila*	69.76	69.96	74.23	**2,357**	1,224
*Arthrobacter arilaitensis*	68.57	68.04	69.35	69.05	**3,436**

^†^Upper right, numbers of orthologous proteins shared between genomes; lower left, AGIOS values; bold numbers indicate the numbers of proteins per genome.

## Genomic comparison

We compared the genome of *N. massiliensis* strain NP1^T^ to those of *N. alba* strain DSM 19423, *M. luteus* strain NCTC2665, *K. rhizophila* strain DSM 2048 and *A. arilaitensis* strain RE117. The draft genome of *N. massiliensis* had a larger size than those of *N. alba* and *M. luteus* (2.72, 2.59 and 2.7 Mb, respectively) but was smaller than that of *A. arilaitensis* (3.92 Mb). The G+C content of *N. massiliensis* was lower than those of *N. alba, M. luteus* and *K. rhizophila* (62.4, 63.8, 73 and 71.2%, respectively) but higher than that of *A. arilaitensis* (59.3%). The gene content of *N. massiliensis* was larger than those of *N. alba, M. luteus* and *K. rhizophila* (2,714, 2,403, 2,345 and 2,414 genes, respectively) but smaller than that of *A. arilaitensis* (3,771). In addition, *N. massiliensis* shared 1,142, 1,132, 1,087 and 1,208 orthologous genes with *N. alba, M. luteus* and *K. rhizophila*, respectively. *N. massiliensis* exhibited an AGIOS value of 75.61% with *N. alba*, and 68.57 to 70.5 with other members of the family *Micrococcaceae*.

## Conclusion

On the basis of phenotypic, phylogenetic and genomic analyses, we formally propose the creation of *Nesterenkonia massiliensis* sp. nov. that contains the strain NP1^T^. The strain has been isolated from the fecal flora of an AIDS-infected patient living in Marseille, France. Several other bacterial species were also cultivated from different fecal samples through diversification of culture conditions [4-34], thus suggesting that the human fecal flora of humans remains only partially known.

### Description of *Nesterenkonia massiliensis* sp. nov.

*Nesterenkonia massiliensis* (mas.si.li.en′sis. L. gen. fem. n. massiliensis of Massilia, the Roman name of Marseille, France, where the type strain was isolated).

Grows between 25 and 45°C on blood-enriched Columbia agar (BioMerieux). Optimal growth obtained at 37°C in aerobic atmosphere. Weak growth in microaerophilic atmosphere. No growth under anaerobic condition. Colonies are dark yellow and 1 mm in diameter. Cells are Gram-positive, non-endospore-forming, and motile cocci, with a mean diameter and length of 0.67 μm and 1.4 μm, respectively. Catalase positive, oxidase negative. Negative reactions obtained for nitrate reduction, urease, indole production, glucose fermentation, arginine dihydrolase, β-galactosidase, glucose, arabinose, mannose, mannitol, N-acetyl-glucosamine, maltose, gluconate, caprate, adipate, malate, citrate, phenylacetate assimilation and cytochrome oxidase. Fermentation of D-glucose, D-fructose, D-saccharose, ribose, mannose, mannitol, D-trehalose and L-rhamnose. No reaction observed for esculin, salicin, D-cellobiose and gentiobiose. Cells are susceptible to amoxicillin, imipenem, rifampin, ciprofloxacin, gentamicin, doxycycline and vancomycin but resistant to trimethoprim/sulfamethoxazole and metronidazole.

The G+C content of the genome is 62.47%. The 16S rRNA and genome sequences are deposited in GenBank under accession numbers JX424770 and CBLL00000000, respectively. The habitat of the microorganism is the human digestive tract. The type strain NP1^T^ (= CSUR P244 = DSM 26221) was isolated from the fecal flora of a 32-year-old French female suffering from AIDS.
